# Perforated appendicitis with endosalpingiosis

**DOI:** 10.1093/jscr/rjae091

**Published:** 2024-02-28

**Authors:** Andrew J Sealey, Janaka Balasooriya

**Affiliations:** General Surgery, Canberra Health Services, Yamba Drive, Acton 2605, Australia; General Surgery, Canberra Health Services, Yamba Drive, Acton 2605, Australia

**Keywords:** appendicitis, perforation, endosalpingiosis, malignancy

## Abstract

Endosalpingiosis a condition of ectopic glandular epithelium diagnosed histologically, most commonly on pelvic and abdominal peritoneum, that can be associated with abdominal pain mimicking appendicitis. There is evidence emerging that endosalpingiosis may be associated with serous ovarian malignancies. Here we describe a case of perforated appendicitis with concurrent endosalpingiosis. Further research is required to better elucidate the association between endosalpingiosis and malignancy, and the implications of a concurrent presentation with a hollow viscus perforation.

## Introduction

Endosalpingiosis is a rare histopathological diagnosis of ectopic, bland glandular epithelium with ciliated tubular cells considered part of the spectrum of peritoneal serous lesions. The incidence is difficult to determine as it is only diagnosed through biopsy; however, one prospective study on 1107 women undergoing a laparoscopy found that 7.6% had evidence of endosalpingiosis [1]. Forty percent of women with endosalpingiosis were post-menopausal, and 34% had concurrent endometriosis. It might be more frequent in women who have had previous gynaecologic procedures, and may be associated with pain, infertility, a pelvic mass, and/or urinary symptoms [2]. Previous studies have reported endosalpingiosis involving the peritoneum, pelvic organs, retroperitoneum, skin, spinal nerve root attachment, axillary lymph nodes, and the appendix [3–7]. Most cases of appendiceal endosalpingiosis reported are in a benign appendix; to date, there is only one case report of endosalpingiosis in acute appendicitis, without perforation [8]. Here we report a case of perforated appendicitis with endosalpingiosis.

## Case report

**Figure 1:**
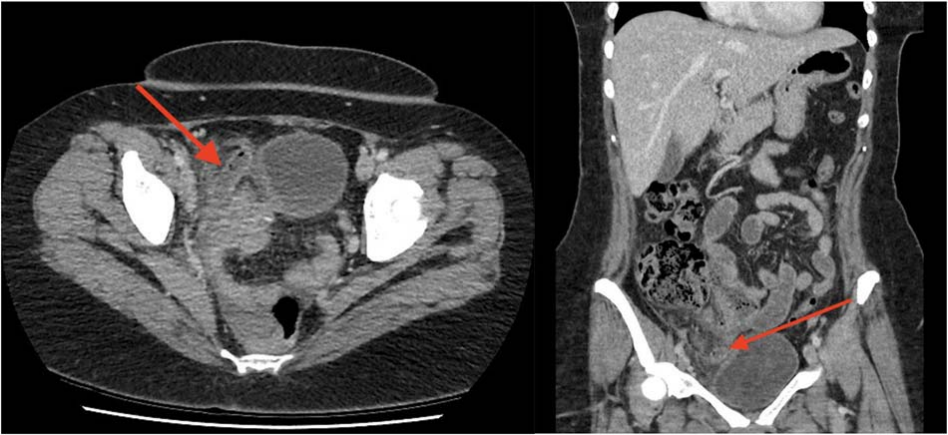
Axial (a) and coronal (b) images from portal-venous phase CT of the abdomen and pelvis demonstrating acute appendicitis. Arrow to inflamed, enlarged appendix.

A woman in her 40s presented to a nearby district hospital with 1 day of abdominal pain. Pain was initially a dull ache in her right upper quadrant radiating to her back, she had no other associated symptoms. After review, she was discharged with a referral for an abdominal ultrasound to look for a biliary cause. Later that afternoon, pain became severe, migrating to her lower abdomen and she represented. There were no bowel or urinary symptoms. The patient was usually fit and well and took no regular medications. In her past, she had severe menorrhagia and dysmenorrhea for which she had an endometrial ablation, and later a transvaginal total hysterectomy. She described her pain as identical to the severe pain she used to have with menstruation. Her white cell count was 20.4 × 10^9^ cells/L and C-reactive protein 378.9 mg/L; biochemistry and liver function tests were otherwise unremarkable. A CT was performed that revealed a 14-mm appendix with significant surrounding fat stranding and a small volume of free fluid (Fig. 1). There was no free gas or organized collection. The patient was then accepted for transfer to our hospital’s emergency department. Upon arrival, she was haemodynamically stable and afebrile, her abdomen revealed generalized lower abdominal tenderness with focal peritonism in the right iliac fossa.

A laparoscopic appendicectomy was performed. Intra-operatively the appendix was perforated, there were multiple free floating faecoliths, interloop abscesses, and a reactive small bowel ileus. The ovaries were macroscopically normal. There were no operative complications, a 15Fr Blakes drain was placed in the pelvis and the patient continued intravenous antibiotics post-operatively. The patient’s post-operative course was uneventful, the drain was removed on Day 3, and the patient discharged on Day 4 with a course of oral antibiotics. Histopathology revealed gangrenous acute appendicitis and occasional glands lined by bland epithelium with ciliated, flattened to columnar cells consistent with endosalpingiosis. There was no evidence of epithelial dysplasia or neoplasia.

## Discussion

Endosalpingiosis is categorized under the umbrella of peritoneal serous lesions. Typical histological features are benign glands lined by ciliated tubal-type epithelium resembling that of the endosalpinx, unlike endometriosis there is no endometrial stroma.

The diagnosis is pathological and the clinical significance of endosalpingiosis remains unclear. A study of 51 patients with chronic pelvic pain undergoing laparoscopy found that six had endosalpingiosis, but four had concurrent endometriosis [9]. A retrospective review of 13 patients found that five were asymptomatic, five had abdominal or pelvic pain, but only in four did the localisation of endosalpingiosis correlate with their pain. However, pelvic adhesions were the most prevalent finding and the authors concluded endosalpingiosis as incidental rather than causal [10].

Other than pain, there are studies that suggest an association between endosalpingiosis and gynaecologic malignancies. One study on 2490 women with endosalpingiosis reported an incident rate ratio of 43.7% for ovarian cancers [11]. A separate study reported that 40% of patients with endosalpingiosis had concurrent malignancy, the majority were of gynaecological origin, and less frequent were breast and colorectal cancers [12]. Further studies are required to better appreciate this association.

Endosalpingiosis of the appendix is remarkably rare with only a small number of cases published in the literature, most of which are in an otherwise benign appendix. To our knowledge, there is only one published case report of endosalpingiosis in acute uncomplicated appendicitis, and no reports in complicated appendicitis. Here we have reported on a second case in appendicitis, complicated by perforation in a woman who has had prior gynaecologic surgery for benign pathology. To date, there are no studies reporting on an association between appendiceal endosalpingiosis and gynaecological malignancies in appendicitis with or without perforation. The clinical significance of a perforation and development further peritoneal endosalpingiosis or malignancy is entirely unknown. Accordingly, there are no guidelines regarding post-operative surveillance. In our patient, we have recommended routine breast screening, and a symptom-based approach to further investigation and follow-up for ovarian malignancies as there is insufficient evidence for screening. A referral for colonoscopy has been recommended as patients with appendicitis are at increased risk of colorectal cancer [13, 14], even in the absence of endosalpingiosis.

## Conclusion

Endosalpingiosis is a rare pathology diagnosed on histology most commonly in post-menopausal women, and women who have had prior gynaecologic surgery. Its clinical significance is not fully understood; however, it may be associated with gynaecologic malignancies. We have presented a case of endosalpingiosis in a perforated, gangrenous appendicitis and highlighted the need for further research.
